# Exploring the full potential of sperm function with nanotechnology tools

**DOI:** 10.1590/1984-3143-AR2024-0033

**Published:** 2024-08-16

**Authors:** Serge Leugoué Kameni, Notsile Hleliwe Dlamini, Jean Magloire Feugang

**Affiliations:** 1 Mississippi State University, Department of Animal and Dairy Sciences, Mississippi State, MS, USA

**Keywords:** biomarkers, functional genomics, nanoparticles, spermatozoa

## Abstract

Sperm quality is essential to guarantee the success of assisted reproduction. However, selecting high-quality sperm and maintaining it during (cryo)preservation for high efficiency remains challenging in livestock reproduction. A comprehensive understanding of sperm biology allows for better assessment of sperm quality, which could replace conventional sperm analyses used today to predict fertility with low accuracy. Omics approaches have revealed numerous biomarkers associated with various sperm phenotypic traits such as quality, survival during storage, freezability, and fertility. At the same time, nanotechnology is emerging as a new biotechnology with high potential for use in preparing sperm intended to improve reproduction in livestock. The unique physicochemical properties of nanoparticles make them exciting tools for targeting (e.g., sperm damage and sexing) and non-targeting bioapplications. Recent advances in sperm biology have led to the discovery of numerous biomarkers, making it possible to target specific subpopulations of spermatozoa within the ejaculate. In this review, we explore potential biomarkers associated with sperm phenotypes and highlight the benefits of combining these biomarkers with nanoparticles to further improve sperm preparation and technology.

## Introduction

Routine semen analysis, such as sperm motility and morphology, has been used as the main criteria for monitoring sperm quality and subsequent fertility. However, ejaculates approved by these criteria do not necessarily have high fertility (Avendaño et al., 2009; Ferrigno et al., 2021), and there is a need for robust biomarkers to effectively predict fertility and reduce/prevent losses associated with infertility. Functional genomics, a technique capable of describing the functions and interactions between genes, proteins, and metabolites, has allowed the identification of several biomarkers associated with sperm phenotype, such as motility level, fresh, chilled, frozen, or fertility status. Numerous high-throughput technologies have been used to identify biomarkers related to sperm phenotypes, such as fertility and freezability (Peddinti et al., 2008; Soggiu et al., 2013; Menezes et al., 2019; Mateo-Otero et al., 2023; Song et al., 2023; Sun et al., 2023). These potential biomarkers offer novel perspectives for sperm preparations using nanoparticles.

**Figure 1:**
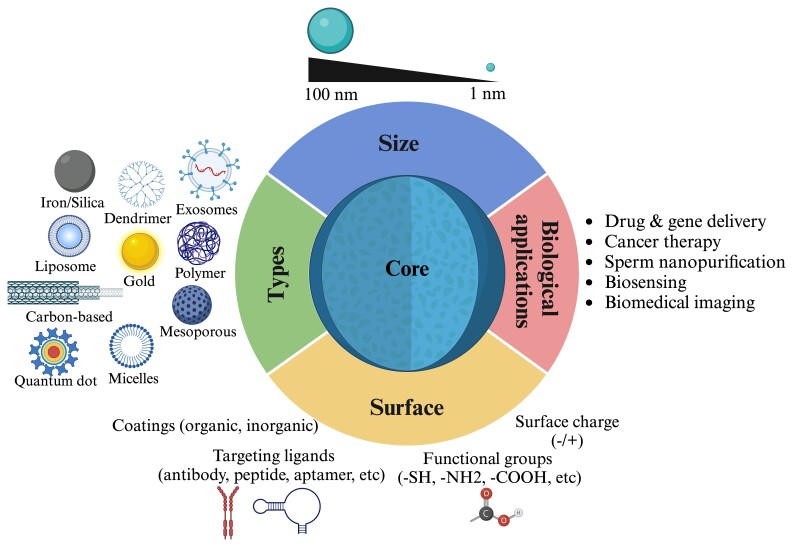
Structure of a typical nanoparticle. Metal nanoparticles (MNPs) have a metal core and a shell. The core can be made of an inorganic metal (zinc, iron, silver, gold, etc.) or metal oxide (Aluminum, copper, magnesium, titanium, zinc, silica, iron oxides, etc.). The shell can be made of an organic (e.g., polymers) or inorganic material (e.g., gold), or metal oxide (e.g., silane). Nanoparticles made of metallic cores surrounded by oxide shells, also known as metal oxide core-shell nanoparticles, have become popular due to their unique properties and high stability. Diverse metal-oxide interactions in metal oxide core-shell nanoparticles enable tuning their electronic structure (shape and size), spectroscopic properties, and surface reactivity for bioapplications (e.g., sensing, drug/gene delivery, and targeted imaging and selection).

Nanoparticles are nanoscale compounds produced naturally by cells as extracellular vesicles or manufactured through bottom-up (synthesis from atoms and molecules) and top-down (synthesis from bulk materials) approaches, possessing various physicochemical properties (e.g., electrical, optical, and magnetism). A typical structure of a nanoparticle with specific properties (e.g., magnetism and fluorescence), mainly acquired from its core composition is shown in Figure1. The US National Nanotechnology Initiative describes nanotechnology as the understanding and controlling matter at the nanoscale, at dimensions between approximately 1 and 100 nanometers, where unique phenomena enable novel applications (NNI, 2024). Nanotechnology has sparked as one of the emerging research fields during the last decades, with bioapplications in human healthcare as diagnostic and therapeutic agents (Abedin et al., 2021; Anjum et al., 2021). This technology holds great potential in veterinary medicine and livestock farming in enhancing animal health, (re)production, and husbandry, acting as antimicrobials, animal growth and well-being promoters (nano-additives in feed), vaccines, and nanomaterials for drug delivery (Hill and Li, 2017; Selokar et al., 2020; ul Haq et al., 2023; Barwant et al., 2024). Especially in reproduction, nanotechnology has enormous potential in semen technology, as nanoparticles could be used for targeted sperm selection or nanoselection/nanopurification (Odhiambo et al., 2014; Feugang et al., 2015a), imaging, (Feugang et al., 2015b; Jain et al., 2018), sexing (Domínguez et al., 2018), and reduction of damages occurring during (cryo) preservation (Falchi et al., 2018a; Khodaei-Motlagh et al., 2022; Khalique et al., 2023).

From this background, semen has unique attributes that could improve the effectiveness of sperm biotechnologies. This review explores sperm biology and identifies key molecular markers linked to sperm phenotypes, such as motility, storage, freezing, sex, and fertility. It also highlights the benefits of combining these biomarkers with nanotechnology tools to improve sperm fertility and technological outcomes.

## Sperm biology

### Spermatogenesis

The spermatozoon consists of the head, which contains the genetic material (DNA); the middle piece, containing the mitochondria; and the tail, responsible for motility. Spermatozoa are highly specialized, compact, and motile cells originated after spermatogenesis to move to the ampulla region, where it interacts with the oocyte and initiate fertilization process. Spermatogenesis is a species-specific cell differentiation process that occurs in the seminiferous epithelial and is regulated by the hypothalamic–pituitary–testicular axis (Amann, 2008).

Spermatogenesis involves three distinct phases, namely proliferation (spermatocytogenesis), meiosis, and differentiation (spermiogenesis) (Staub and Johnson, 2018).

*Proliferation phase or mitotic divisions*– cell divisions occur in a sequence where chromosomes duplicate, resulting in two daughter cells or primary spermatocytes (or spermatocytes I), maintaining intercellular bridge connections with spermatogonia (types A and B). Spermatocytes I possess equal division of chromosomes (e.g. 2n=46 and 4n centromeres) and cytoplasm (Sharma and Agarwal, 2011).

*Meiotic phase* – individual spermatocytes I move into the adluminal compartment, duplicate DNA, and divide into two haploid secondary spermatocytes (spermatocyte II, with n=23 chromosomes and 2n centromeres) (Alpatov et al., 2014). This second stage (meiosis II) immediately follows the first stage of the meiotic phase (meiosis I), involving chromosomal exchange with divided centromeres and giving rise to four haploid daughter cells (n = 23 chromosomes and 1 centromere) (Sharma and Agarwal, 2011).

*Spermiogenesis* – sperm cells undergo complete differentiation or morphogenesis to become highly specialized spermatozoa with compacted chromatin (Durairajanayagam et al., 2015). Numerous nuclear and cytoplasmic changes occur in spermatozoa during that phase. For example, nuclear histone is replaced with protamine to form well-developed disulfide bonds (Holstein et al., 2003), various organelles of the cytoplasm such as the Golgi apparatus, acrosomal cap, proximal centriole, and flagellum structures, go through profound remodeling and reorganization (Cooper, 2005). The midpiece surrounded by a sheath, axial core, and coarse fibrils (Neto et al., 2016) has a high concentration of mitochondria, which are responsible for aerobic functioning and supply energy for sperm motility (Gadella and Luna, 2014). After successive stages of differentiation, the resulting spermatozoa emerge as immature and immotile cells, unable to fertilize the oocyte (Jones, 2002). Produced spermatozoa often carry residual bodies or excess cytoplasm (cytoplasmic droplets) that are phagocyted by surrounding Sertoli cells. This excess of cytoplasm ensured synchronization of the syncytium of spermatids (Gadella and Luna, 2014), maintaining cross communications/bridges among sister spermatids.

### Sperm maturation

The maturation process of spermatozoa is a fascinating journey, intricately influenced by the testosterone. The study of sperm maturation has sparked a growing interest among researchers, leading to a continuous generation of knowledge in this field (Aitken and Baker, 2008; Holt and Morrell, 2013). This process consists of number of changes:

*Morphological changes* – The excess of cytoplasm in produced spermatozoa is eliminated during the journey within the epididymis. Disruptions in epididymal and/or testicular function can lead to sperm abnormalities, such as cytoplasmic droplets and/or abnormally condensed chromatin. A negative correlation between such abnormalities and fertility has been reported (Sutovsky and Lovercamp, 2010).

*Biochemical changes* – During epididymal transit, sperm gain motility and fertilizing potential (Sullivan et al., 2005). Biochemical changes alter the sperm plasma membrane’s biophysical properties, which is fundamental for the occurrence of subsequent steps providing fertilization capacity to the sperm (Holt and Morrell, 2013). Numerous studies have reported the critical effects of post-gonadal sperm maturation on the sperm cell acquiring complete functionality characteristics (Dacheux and Dacheux, 2014). Epididymal epithelial cells secrete nano- to micro-size vesicles, known as epididymosomes, that contain lipids, proteins, and RNAs (Sullivan et al., 2005; Sharma et al., 2016).

*Functional changes* – Within the epididymis, the epididymosome vesicles fuse with sperm membranes to induce surface changes and intracellular delivery of their contents, contributing to sperm functionality acquisition (e.g., motility and fertilization ability). Furthermore, during ejaculation, mature epididymal sperm interacts with several molecules contained in secretions from seminal vesicle glands, prostate (prostasomes), bulbourethral glands, and Cowper glands (Rodríguez-Martínez et al., 2011) to further stabilize the sperm membrane by coating their surface (Lu et al., 2011) and protect them during transit through the female genital tract (vagina, cervix, and utero/oviduct) (Gibbons et al., 2005; Troedsson et al., 2005). More specifically, the prostate gland secretions include the prostasome vesicles, whose lipids, proteins, RNA, and DNA contents also affect sperm function by stimulating motility, regulating capacitation, and protecting against the immune response in the female tract (Aalberts et al., 2014).

## Sperm transit within the female reproductive tract

After deposition within the female reproductive tract, spermatozoa that reach the oviduct exhibit good progressive motility, adequate morphology, normal sperm head and size, sensitivity to signaling molecules, and a normal DNA status (Holt and Fazeli, 2015). In the oviduct, epithelial cells and local fluid play a crucial role in modulating sperm function (e.g., capacitation, acrosome reaction, and fertilization) and local gene expression (López-Úbeda et al., 2015).

### Sperm capacitation

Capacitation is a process that involves biochemical and physiological modifications on the sperm plasma membrane during the passage of sperm within the female reproductive tract (Gadella and Luna, 2014). This process includes the efflux of cholesterol from the sperm plasma membrane, leading to increased permeability to bicarbonate and calcium ions, and changes in protein phosphorylation. Bicarbonate (HCO^3-^) and calcium ions (Ca^2+^) play crucial roles in destabilizing the plasma membrane through the cAMP-dependent protein phosphorylation-signaling pathway (Tardif et al., 2001). The activation of soluble adenyl cyclase generates cyclic adenosine monophosphate (cAMP), in which the calcium ion acts as a secondary messenger, initiating a cascade of protein phosphorylation that triggers sperm motility (Chen et al., 2000; Finkelstein et al., 2020). These controlled alterations activate hyperactive motility and the ability of sperm to undergo an acrosomal reaction when they reach the oocyte (Travis and Kopf, 2002). Overall, the capacitation process could be summarized as successive steps of 1.) acrosome membrane destabilization, 2.) chemical changes in the sperm tail, 3.) increased permeability to Ca^2+^, and 4.) sperm hyperactivation.

*In vivo Capacitation –* Sperm capacitation occurs in the oviduct, where sperm cells shed adhering decapacitation factors and interact with ciliated cells of the oviduct epithelium, but mainly with substances present in the oviduct fluids (e.g., glycosaminoglycans). These sperm cells progress towards the ampullary-isthmic junction, where the ovulated oocyte arrests for fertilization (Sostaric et al., 2008; Talevi and Gualtieri, 2010).

*In vitro Capacitation* – Is induced in a medium containing calcium, bicarbonate ions, and serum albumin, mimicking the ionic and metabolic composition of oviductal fluid (Touré, 2019). Other substances such as cAMP, caffeine, procaine, heparin, progesterone, and methyl β-cyclodextrin are often used for *in vitro* capacitation, a process involving sperm collection from a male donor, their washing in a buffered solution, then incubation in a capacitation medium to initiate capacitation through the synergistic effects of bicarbonate and calcium for boar sperm (Tardif et al., 2001) or negatively charged glycoconjugates for other livestock animals, such as ruminants (Ka̧tska-Ksia̧zkiewicz et al., 2004). Sperm preparation for *in vitro* fertilization (IVF) consist of their selection (Percoll or swim-up) followed by incubation with matured oocytes in IVF media containing substances that trigger the capacitation process (Henkel and Schill, 2003; Lacalle et al., 2022).

### Acrosome reaction and fertilization

The interaction between proteins from the sperm surface and the zona pellucida receptors facilitates the acrosomal reaction. Then, the acrosome-reacted sperm enters the zona pellucida, reaches the perivitelline space, and fuses with the oocyte membrane to transmit the sperm head contents (e.g., DNA, RNA, and protein) into the ooplasm (Coy et al., 2008). Acrosome reaction is controlled by SNARE complexes, leading to the exocytosis of acrosomal contents upon fusion of the plasma membrane with the outer acrosomal membrane. Many studies have used various advanced technologies (e.g., gene silencing, omics) to study critical molecules (e.g., IZUMO1, TMEM95, SOF1, and SPAC6) playing critical roles in sperm-oocyte membrane interaction and fusion (Siu et al., 2021). The functional genome of the spermatozoon, assessed via high-throughput screening methods (omics) has identified numerous sperm molecules that modulate oxidative phosphorylation, cAMP signaling, and sperm-egg interaction (Johnston et al., 2005; Baker et al., 2007; Hitit and Memili, 2022). As such, biomarkers comprising proteins, mRNAs, lipids, and metabolites hold substantial promise in predicting sperm quality and fertility. These biomarkers have the potential to provide a deep understanding of male reproductive health and sperm phenotype.

## Enhancing sperm manipulation through nanotechnology

Screening technologies have revealed specific molecules that are integral to the molecular pathways of sperm biology, influencing various sperm phenotypes (e.g., quality, fertilizing potential, preservation, sex) and male fertility outcomes (Table 1) (Suchocki and Szyda, 2015; Zeng et al., 2021; Dlamini et al., 2023). The unique properties of nanoparticles, such as their small size and high surface area, hold immense potential for their diverse applications in livestock, impacting sperm quality and fertility outcomes (Feugang, 2017; Feugang et al., 2019) (Figure 1).

**Table 1:** Candidate biomarkers of sperm phenotypes for nanotechnology applications.

**Phenotype**	**Sources**	**Biomarker candidates**	**Animals**	**References**
Motility	Sperm	**Proteins**:	Rams	Zhu et al. (2020)
		Phosphatidylethanolamine binding protein 4, Spermatogenesis associated 18, Carboxypeptidase, Acrosin		
		**Proteins:**	Horses	Gaitskell-Phillips et al. (2021a)
		Mannosidase alpha class 2C member 1, Ubiquinone 1 alpha sucomplex subunit 9-like protein, isoleucyl-tRNA synthetase2, mitochondrial acethyl-CoA acetyl transferase 1, Latherin, Ubiquitin-specific peptidase 43		
		**Proteins:**	Horses	Gaitskell-Phillips et al. (2021b)
		Hexokinase 1, Aconitase hydratase mitochondrial, Phosphoinositide phospholipase C, Elongation factor Tu, F actin capping protein subunit alpha		
		**Metabolites:**	Bucks	Yuan et al. (2023a)
		Butyric acid, 1-(2-Methoxy-13-methyl-pentadecanyl)-sn-glycero-3-phosphoetanolamine, 2-O-benzoyl-D-glucose, Trehalulose, Glutamylphenylalanine, Vulgaxanthin-I		
		**Transcripts:**	Bulls	Ganguly et al. (2013)
		PRM1 mRNAs		
Liquid preservation ability/Freezability		**Proteins**: NUDFB8, SDHC, PDIA4, HSPB1	Bucks	Sun et al. (2023)
		**Proteins:**	Boars	Song et al. (2024)
		GPX5, GLRX, ENO4, QPCT, BBS7, OXR1, DHRS4, AP2S1		
		**Metabolites:**	Boars	Zhang et al. (2023)
		Oleic acid, Oleamide, N8-acetylspermidine		
		**Metabolites:**	Boars	Torres et al. (2022)
		Inosine, Hypoxanthine, Creatine, ADP, Niacinamide, Spermine, 2-methylbutyrylcarnitine		
		**Metabolites:**	Boars	Sui et al. (2023)
		L-citruline		
		**Transcripts:**	Boars	Fraser et al. (2020)
		FOS, NFATC3, EAF2, BAMBI, PTPRU, PTPN2, ND6, ACADM,		
Fertility		**Proteins**: Calmodulin,	Bulls	Soggiu et al. (2013)
		ATP synthase mitochondrial subunits alpha and delta, Malate dehydrogenase and Sperm equatorial segment protein 1		
		**Proteins:**	Boars	Kwon et al. (2015)
		Ras-related protein Rab-2A, Cytochrome b-c1 complex subunit 1, Cytochrome b-c1 complex subunit 2		
		**Proteins**: Acyl-CoA thioesterase 9, Albumin, Casein kinase 2, K voltage-gated channel shaker-related	Bulls	Peddinti et al. (2008)
		**Proteins:**	Bulls	Zhang et al. (2021)
		L amino acid oxidase 1		
		**Metabolites**:	Bulls	Menezes et al. (2019)
		Gamma-aminobutyric acid, Carbamate, Benzoic acid, Lactic acid, Palmitic acid		
		**Metabolites:**	Bulls	DasGupta et al. (2021)
		Taurine, Hypotaurine		
		**Transcripts:**	Bulls	Lalancette et al. (2008)
		rRNA genes (18S, 12S, and Large chain R)		
Mitochondrial activity		**Proteins**:	Stallions	Gaitskell-Phillips et al. (2021b)
		Phosphoglycerate mutase, peroxiredoxin 6-like proteins, actin-1 analogue, transmembrane protein analogue		
Viability		Chaperonin TCP1 subunit 8, Testis expressed 101	Stallions	
Motility	Seminal plasma	**Proteins:**	Bucks	Jia et al. (2021)
		Zonadhesin, Superoxide dismutase, Sperm equatorial segment protein 1, Mitochondrial thioredoxin reductase, Zona pellucida binding protein, Aquaporin 7		
		**Metabolites:**		
		Thioetheramide-PC, Adenosine, N,N-Dimethylguanosine, Isocitric acid		
		**Transcripts:**	Boars	Zhao et al. (2024)
		miRNAs (ssc-miR-		
		122–5p, ssc-miR-486, ssc-miR-451, ssc-miR-345–3p, ssc-miR-362, and ssc-miR-500–5p)		
Liquid preservation ability/Freezability		**Metabolites:**	Bulls	Pessoa et al. (2023)
		Propanoic acid, D-ribose, glycine		
		**Metabolites:**	Boars	Song et al. (2023)
		D-proline, Arginine, L-citruline, Phenylalanine, Leucine, DL-proline, DL-serine, Indole		
		**Metabolites:**	Boars	Sui et al. (2023)
		Tryptophan		
Fertility		**Proteins:**	Boars	Zeng et al. (2021)
		Ceruloplasmin, Carboxypeptidase E, Serine protease inhibitor family A member 12		

### Nanoparticles

**Figure 2:**
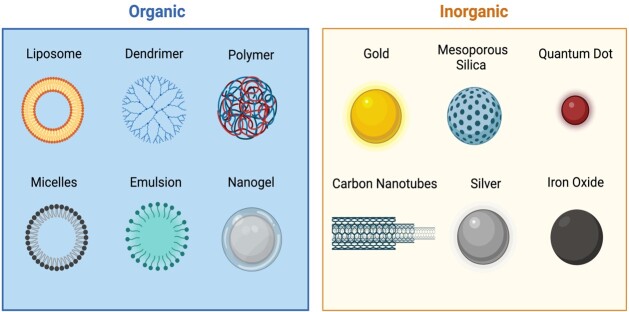
Overview of commonly used inorganic and organic nanoparticles. The presented inorganic nanoparticles can be coated with single or multiple layers of organic or inorganic materials before biofunctionalization for bioapplications. Among the organic nanoparticles, liposomes are the most widely used for bioapplications, such as targeted bioimaging, drug delivery (hydrophobic and hydrophilic), and drug and nucleic acid intracellular delivery. The possibility of coating the external surface of nanoparticles with polyethylene glycol (PEG), or PEGylation, increases nanoparticle’s stability and decreases immunogenicity during *in vivo* delivery.

Nanoparticles can be naturally or artificially produced in various shapes (such as cubic or spherical) and can consist of inorganic (like metals or salts) as well as organic materials (e.g., lipids, proteins or polymers) (Figures 1 and 2) (Sun et al., 2014). Metal nanoparticles are made in different sizes (ranging from 1 to 100 nm) and shapes (like irregular, rod, spherical, cylindrical, tetragonal, and hexagonal) using inorganic core materials such as cadmium, zinc, gold, silver, platinum, plomb, aluminum, nickel, iron, and copper. The composition of these core materials determines their fundamental properties, like electronical, optical, and physical characteristics, which have garnered much attention in medicine. However, these core materials are often associated with immunogenicity and cytotoxicity of nanoparticles because they can release metallic ions like cadmium (Han et al., 2016; Jain et al., 2018; Kuo et al., 2017; Wang et al., 2020). Nonetheless, coating the core material with multiple layers has helped mitigate its toxicity. For instance, the stabilization of the core material with an inorganic protective shell (e.g., zinc sulfide and silica) and surface modification of the core-shell with various polymers (such as polyvinylpyrrolidone - PVP, polyvinyl alcohol - PVA, polyethylene glycol - PEG, among others), ceramics (like Silicates), and adsorption of anions or charged groups (such as citrate^3-^, chloride ions-Cl^-^, dextran sulfate, polyethyleneimine, etc.) reduces nanoparticle toxicity and enhances biocompatibility (particle dispersion in biofluids), which is essential for bioapplications (Suk et al., 2016; Jain et al., 2018).

Organic nanoparticles (e.g., micelle, dendrimer, liposome, nanogel, and polymeric) contrast their inorganic counterparts. They are formed from aggregated molecules (e.g., polysaccharides and lipids) or polymers of various structures, each exhibiting unique size-dependent physical and chemical properties (e.g., optical and electrochemical). The non-toxic and biodegradable micelles and liposomes, with their hollow spheres, are widely used for pharmaceutical transportation (e.g., drugs and nucleic acids). These organic nanoparticles are designed for bioapplications, and their surface modification with polymers (e.g., PEG) often provides an extended lifespan (Suk et al., 2016; Virlan et al., 2016; Feugang et al., 2022).

**Figure 3:**
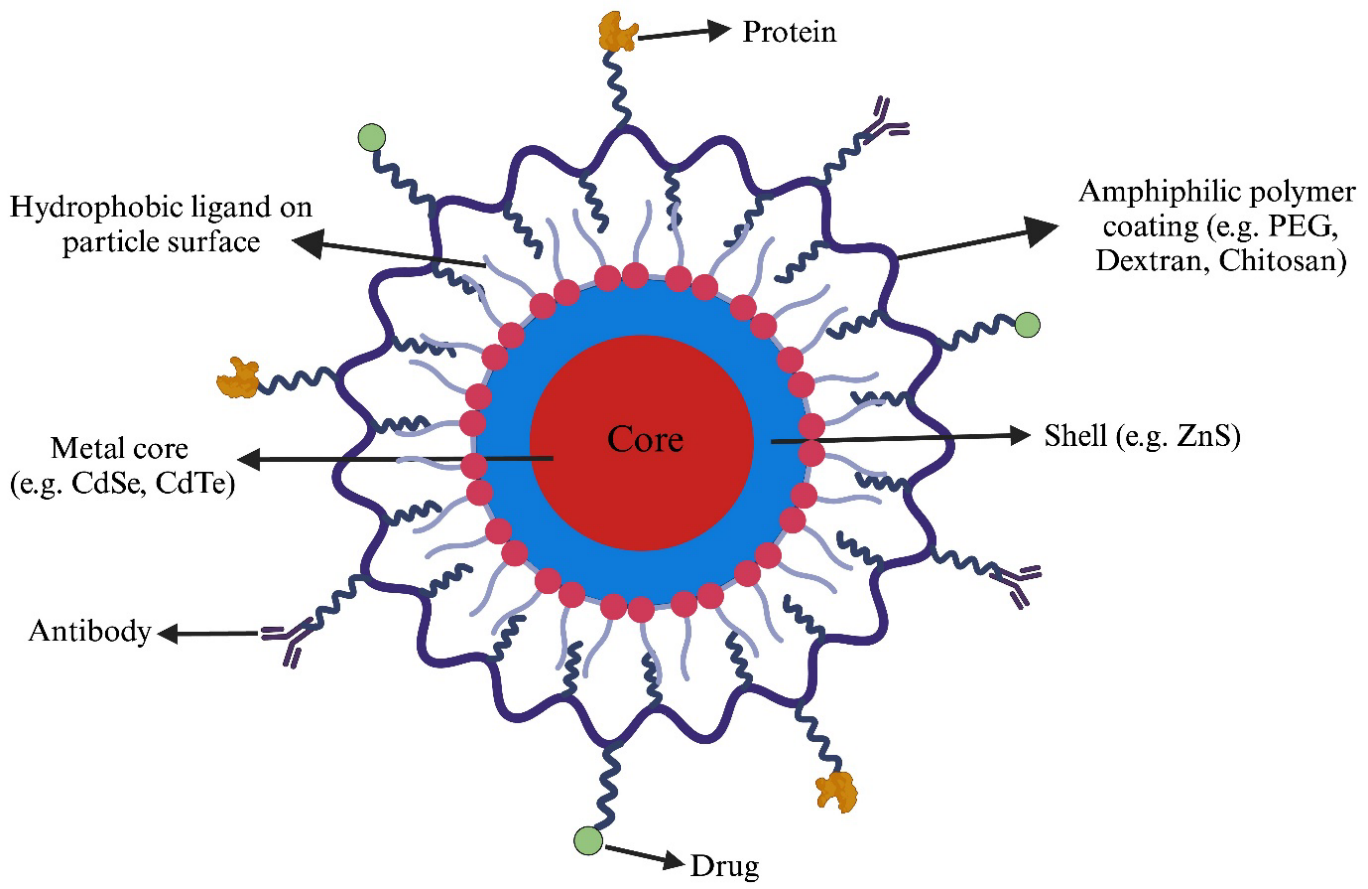
Schematic representation of a biofunctionalized nanoparticle. Ready-to-use nanoparticles for bioapplications include a (nano)core surrounded by single or multiple coating layers providing stability and decreasing immunogenicity and an outer surface layer functionalized based on planned unimodal (e.g., imaging) or multimodal (e.g., imaging and drug administration) applications.

Inorganic and organic nanoparticles are biofunctionalized with attached bioactive ligands such as antibodies, oligonucleotides, peptides, and drugs to their external coating layer, providing specificity in cell targeting (Table 2 and Figure 3) (Feugang et al., 2015b; Jain et al., 2018). The high surface area-to-volume ratio and size-dependent physicochemical properties of both inorganic and organic nanoparticles make them versatile and exciting for a wide range of sperm manipulation (Table 2), such as high visibility/contrast in multiple bioimaging, biocompatibility and dispersibility, specific and molecular targeting, and ability to load and deliver diverse cargos for controlled release to target cells (Feugang, 2017; Sutovsky et al., 2024).

**Table 2:** Examples of nanoparticles for sperm manipulation.

**Nanoparticles**				**Biological applications**	**References**
**Classes**	**Types**	**Shape**	**Size (nm)**		
Organic	Liposomes	Sphere	50-500	Active tumor targeting, drug gene delivery, intracellular delivery, sperm cryopreservation.	Röpke et al. (2011); Deshpande et al. (2013); Mo et al. (2014); Ansari et al. (2016); Feugang et al. (2022)
	Exosomes	Sphere	40 to 120	Sperm function, immune response, cancer therapy, drug delivery	Montecalvo et al. (2012); Du et al. (2016); Fitts et al. (2019)
	Carbon-based: Single-walled (SWCNT)/Multi-walled (MWNT)	Nanotubes	1-200	Gene delivery, sensing, pathogenicity, oxidative stress, inflammation	Donaldson et al. (2006); Donaldson and Tran (2002)
	Polymeric: Nanocapsules/nanospheres (polylactides, polylactide-co-glycolide, chitosan, albumin, gelatin)	Sphere	1-1000	Drug delivery, theragnostics, bioimaging	Szczęch and Szczepanowicz (2020); Zielińska et al. (2020)
Inorganic	Metal nanoparticles: e.g. Gold (Au)	Sphere	2-250	Biomedical imaging, photothermal therapy	Paciotti et al. (2006); Yang et al. (2019)
	Metal oxide nanoparticles: e.g. Fe_2_O_3,_ CeO_2_	Sphere	15-60	Sperm nanopurification, drug delivery vehicles, thermal-based therapy, sperm bio-imaging	Feugang et al. (2015ª); Falchi et al. (2018a) ; Arias et al. (2018)
	Non-metal oxide nanoparticles: e.g. Silica (Si)	Sphere	40-100	Purification, gene and drug delivery, biomedical imaging	Wang et al. (2008); Xu et al. (2019)
	Quantum dots	Sphere	1-10	Drug delivery, photodynamic therapy, biomedical imaging, biosensing	Kim et al. (2004); Weng and Ren (2006); Bera et al. (2010); Feugang et al. (2012)

### Sperm phenotype and nanotechnology applications

#### Sperm motility, morphology, and fertility

Sperm motility and morphology are the golden standard for semen evaluation, serving as selection criteria for further processing of ejaculates post-collection. Motility assessment is conducted objectively with automated systems such as computer-assisted sperm analyzer, which allows evaluation of motility and a wide range of kinematic parameters. This assessment, encompassing a diverse range of livestock species, has contributed to establishing a positive correlation between motility parameters and fertility. The research conducted using both fresh or cryopreserved semen (Ansari et al., 2016; Elmi et al., 2018; Lucca et al., 2021; Khan et al., 2024), has consistently shown that high-fertile males tend to exhibit superior total and progressive sperm motility and velocity parameters than their low-fertile counterparts (Vicente-Fiel et al., 2014). These findings suggest that the differential fertility outcomes may be influenced by the morphology and other defects of spermatozoa.

The percentage of morphologically normal spermatozoa is positively correlated with pregnancy rates in all species (Cecere, 2014). On the other hand, sperm defects, such as the aplastic midpiece in bull ejaculates, disrupt sperm functions, especially motility (Díaz-Miranda et al., 2020). Several studies have used proteomics and metabolomics in various livestock species to identify important molecules in sperm associated with morphological defects in the head, midpiece, and tail, which can affect sperm motility and fertility (Peddinti et al., 2008; Menezes et al., 2019; Zhu et al., 2020; DasGupta et al., 2021; Gaitskell-Phillips et al., 2021a; Hitit et al., 2021; Jia et al., 2021; Zeng et al., 2021; Sun et al., 2023). These identified molecules, which may interact with sperm membranes to influence sperm function, can potentially serve as biomarkers for monitoring sperm quality, including motility and morphology. When combining with traditional sperm attributes, these biomarkers may help in selecting the best spermatozoa/ejaculates and predicting fertility more accurately (Sellem et al., 2015; Inanç et al., 2018).

The knowledge of the abovementioned sperm defects with associated biomarkers, such as the lectin and annexin V, targeting damaged acrosome and DNA, respectively, has led to the adoption of different methods of sperm purification using biofunctionalized (or conjugated) nanoparticles to obtain high-quality sperm through targeted elimination of low-quality or immotile sperm in an ejaculate (Feugang, 2017; Meles et al., 2022). Among these techniques, the density gradient and single-layer centrifugation protocols use nanosized compounds such as the polyvinylpyrrolidone-, silane-, and glycidoxypropyltrimethoxsilane-coated silica colloid solutions, commercialized as Percoll^™^, Puresperm^™^, and Androcoll^™^, respectively. These nanoparticle-based solutions separate pure, high-quality, and viable spermatozoa that have shown numerous applications in reproductive technologies (*in vitro* fertilization, intracytoplasmic sperm injection or ICSI, and artificial insemination) of various livestock species.

#### Sperm viability

Defects related to membrane (plasma, acrosome, and mitochondrial) losses, capacitation issues, and DNA fragmentation were collectively classified as sperm viability abnormalities (Sutovsky et al., 2024). Sperm membrane integrity is a core attribute of spermatozoa, whose defects are associated with a decrease in the overall functionality of spermatozoa (motility, survival, acrosome reaction, ova binding), leading to early embryonic loss or genetic diseases (Avendaño et al., 2009; Ferrigno et al., 2021). Plasma membrane defects could be mechanically and/or chemically induced during normal or abnormal physiological processes, leading to damage such as membrane lipid peroxidation and externalization of phosphatidyl serine residues, an indicator of early apoptosis, with subsequent decrease in fertilizing potential (Sutovsky et al., 2024). Acrosome reaction and capacitation are determinant factors of fertilization success that occur during normal physiological conditions, where sperm capacitation is associated with specific markers and molecular pathways related to fertility outcomes (Kwon et al., 2015; Bae et al., 2022). Nevertheless, both acrosome reaction and capacitation processes are affected during semen manipulation (Yoon et al., 2015; Yeste, 2016; Lee et al., 2023). Acrosome-reacted sperm cells exhibit membrane markers that could be targeted for viability evaluation of semen quality. Mitochondrial membrane integrity portrays mitochondria status and energy reserves used by the cell to achieve different physiological functions as a regulator and indicator of sperm motility that could be related to male fertility (Agnihotri et al., 2016; Gallo et al., 2021). Apoptosis and DNA integrity are essential for successfully transmitting the paternal genome, fertilization, and normal embryo development (Kumaresan et al., 2020). Apoptotic spermatozoa are routinely evaluated through Annexin V, which interacts with externalized phosphatidyl serine residues on their plasma membrane. Furthermore, the highly basic protamine 1 protein, participating in DNA packaging, is positively correlated with bull sperm motility (Ganguly et al., 2013) and fertility (Dogan et al., 2015; Llavanera et al., 2021; Souza et al., 2018), with further confirmation in boar spermatozoa (Alvarez-Rodriguez et al., 2021). Interestingly, a recent study demonstrated a positive correlation between DNA fragmentation and protamine deficiency (Kherzi et al., 2019), making protamine a potential target for selecting spermatozoa with high motility and fertility potentials.

The current evaluation methods of semen quality often lead to a binary outcome, categorizing semen as either “Passed” or “Failed” for breeding. Supplementing extenders with organic nanoparticles like loaded or unloaded liposome vesicles and exosomes may reduce the proportion of non-viable sperm cells in semen. Both organic nanoparticles hold promise, demonstrating their potential in repairing damaged spermatozoa through their ability to bind sperm plasma membranes of numerous livestock species (He et al., 2001; Röpke et al., 2011; Pillet et al., 2012; Kumar et al., 2015; Luna-Orozco et al., 2019; Medina-León et al., 2019; Mafolo et al., 2020; Mortazavi et al., 2020). Loaded liposomes and tissular or body-fluids isolated exosomes can be used as nanocarriers for intraspermatic cargo delivery of specific molecules. Unlike the manufactured liposomes that can encapsulate any desired molecules (Feugang et al., 2022) for delivery, exosomes are rich in various molecules (e.g., proteins, miRNA, lipids, metabolites, and mRNA) isolated from various biofluids, including seminal plasma (Piehl et al., 2013; Yang et al., 2017; Dilsiz, 2022; Dlamini et al., 2023). Following binding with spermatozoa, these exosome contents entering spermatozoa may participate in energy pathways, protein metabolism, and maintenance of recipient cells, influencing sperm maturation, capacitation, acrosome reaction, and fertility (Piehl et al., 2013; Du et al., 2016; Qamar et al., 2019). The presence of extracellular vesicles or exosomes in cryopreservation extenders improves post-thaw sperm motility, viability, mitochondrial activity, and membrane integrity (Qamar et al., 2019).

On the other hand, magnetic metal oxide nanoparticles were designed to target damaged cells for removal under a magnetic field to increase the chance of the ejaculated to be approved. In early studies with bovine (Odhiambo et al., 2014) and porcine (Feugang et al., 2015a; Durfey et al., 2019) species, iron oxide nanoparticles were conjugated with lectin, ubiquitin, and annexin V to selectively target and remove acrosome reacted, damaged, and early apoptotic spermatozoa from semen doses without impairing sperm fertility potential.

#### Sperm freezability

Sperm cryopreservation is a process that allows the long-term storage of semen in liquid nitrogen and is an essential technology for preserving animal fertility (Hungerford et al., 2022). Several sperm functions, including DNA fragmentation, early capacitation, and acrosome and membrane integrity, are impaired during cryopreservation (Yoon et al., 2015; Yeste, 2016), reducing the capacity of spermatozoa to achieve successful fertilization. During this process, spermatozoa experience extreme stress at different proteins, DNA, lipid metabolism levels, and long-chain polyunsaturated fatty acids in the plasma membrane, affecting sperm freezability (Ugur et al., 2019).

Studies of metabolite dynamics in sperm have shown signature differences between fresh, chilled, and frozen-thawed buck (Yuan et al., 2023b) and boar (Torres et al., 2022; Zhang et al., 2023) spermatozoa. Abundance in specific metabolites (e.g., propanoic acid, D-ribose and glycine) in seminal plasma is associated with higher liquid storage ability/freezability of spermatozoa (Pessoa et al., 2023; Song et al., 2023; Zeng et al., 2021). Overall, studies indicate that sperm metabolome changes (e.g., lipids, lipid-like molecules, organic acids and their derivatives) during cryopreservation and influence various biological pathways, such as the linoleic acid metabolism pathway (Yuan et al., 2023b). Further, sperm-borne L-citrulline and seminal plasma-derived tryptophan have been proposed as potential sperm freezability markers in boars (Sui et al., 2023). Similarly, numerous studies have reported the beneficial effects of free amino acids (e.g., alanine, glutamine, histidine, and proline), acting as inhibitors of lipid peroxidation or osmotic regulators. Their presence in extenders or detection in semen has been associated with sperm freezability, thereby enhancing post-thaw viability and quality (Atessahin et al., 2008; Saravia et al., 2009; Trimeche et al., 1999; Ugur et al., 2020).

Similarly, numerous proteomic analyses of sperm ejaculates (sperm and seminal plasma) have unraveled biomarkers (Table 1) and molecular pathways associated with high-quality spermatozoa of many livestock species (Zhu et al., 2020; Gaitskell-Phillips et al., 2021a; Hitit and Memili, 2022). For instance, a study revealed hundreds of differentially expressed proteins between low- and high-freezability buck spermatozoa, with several of these proteins associated with various biological pathways influencing freezability (Sun et al., 2023). Other studies have revealed protein markers (e.g., heat shock protein 90 or HSP90, heat shock protein A8 or HSPA8, or lipocalin-type prostaglandin D synthase or L-PDGS) associated with the sperm freezability status (Ugur et al., 2019). These proteins and metabolites can serve as biomarkers to target high-freezability spermatozoa or could be supplemented associated with nanoparticles in extenders (Table 2). The potential of various nanoparticles, including liposomes and exosomes for successful semen cryopreservation have been evocated in previous works, summarized by (Saadeldin et al., 2020).

Liposome vesicle nanoparticles loaded or not with the identified biomarkers can fuse with the sperm plasma membrane and deliver their contents within the sperm cytoplasm to mitigate sperm damage caused by the freezing-thawing process (He et al., 2001; Purdy and Graham, 2015). The addition of liposomes in egg-free commercial extender (e.g., OptiXcell®) attenuates the damages of bull, buffalo (Ansari et al., 2016), and dromedary camel (Al-Bulushi et al., 2019; Swelum et al., 2019) sperm. Similarly, exosomes with specific contents may play a crucial role in repairing damaged sperm during freezing-thawing (Saadeldin et al., 2020). Furthermore, incubation of spermatozoa with exosomes before cooling or post-thawing increases the antioxidant activity of stored spermatozoa, improving motility, viability, mitochondrial activity, and membrane integrity of post-thaw canine (Qamar et al., 2019) and rat (Mokarizadeh et al., 2013) semen, while decreases the levels of ROS and malondialdehyde content. Furthermore, classes of nanoparticles can be used to discriminate high versus low freezability spermatozoa for enhanced fertility. The design of magnetic nanoparticle conjugates to target a population of spermatozoa has shown beneficial, with no toxicity effects, in enriching bovine (Odhiambo et al., 2014), equine (Domínguez et al., 2018), and porcine (Durfey et al., 2019) semen with desired sub-populations.

#### Sperm preservation

Liquid sperm preservation in an appropriate extender is critical for prolonged chilled (15-18^o^C) or cold (4-10^o^C) storage, especially in species with poor freezability spermatozoa such as pigs. Harvested semen are generally mixed with extenders containing various protective and nutritive compounds for spermatozoa survival (Kameni et al., 2021; Wiebke et al., 2022), and maintaining extended semen at low temperatures during storage is crucial to reducing sperm metabolism and ATP production, and detrimental byproducts (e.g., reactive oxygen species or ROS) are routine in breeding studs for sperm performance preservation. However, despite these precautions, spermatozoa still experience harmful effects of metabolic decoupling, ion imbalance, activated proteases, cellular acidosis, energy deprivation, and ROS that gradually accumulate in the medium and weaken spermatozoa through multiple damages (Falchi et al., 2018b; Ugur et al., 2019; Kameni et al., 2021).

ROS are among the many toxins the spermatozoa release in the extender, affecting sperm quality during prolonged preservation. The use of antioxidative nanoparticles, such as cerium oxide and vitamin E nanoemulsions, has shown beneficial effects on ram spermatozoa during chilled preservation (Falchi et al., 2018a; Jurado-Campos et al., 2023) Nonetheless, the different abilities of boar semen in sustaining chilling have permitted the identification of differentially expressed proteins (187) of which several were involved in the defense mechanisms against oxidative stress, assembly and maintenance of sperm motility, and sperm metabolism and capacitation. These subsets of proteins could be considered putative biomarkers of sperm quality or preservation (Song et al., 2024). The identified biomarkers are gold mines for the application of appropriate nanoparticles to discriminate high versus low sperm preservability. There is potential in designing magnetic nanoparticle conjugates to selectively target a population of spermatozoa, thus enriching semen doses with high freezability spermatozoa. Furthermore, the application of magnetic nanoparticle conjugates to target the identified protein marker candidates has potential to contribute to enhanced fertility outcomes of preserved semen (Odhiambo et al., 2014; Feugang et al., 2015a).

#### Semen redox status

Oxidative stress results from the excess of ROS produced during the oxygen metabolism of spermatozoa. To maintain optimal sperm function, a balance between oxidation and reduction is necessary, involving the neutralization of ROS through antioxidants. The antioxidative status has been linked to sperm fertility potential, having beneficial roles at normal levels during acrosome reaction and fusion with the oocyte. Rupture of the redox equilibrium leads to oxidative stress, lipid peroxidation, and subsequent alterations in sperm characteristics and fertility (Gundogan et al., 2010; Sapanidou et al., 2023). Redox disequilibrium is aggravated during post-collection semen manipulation (centrifugation, freezing, thawing, and incubation) causing damage to sperm membranes rich in polyunsaturated fatty acids. Lipid peroxidation has shown a negative correlation with sperm characteristics of bulls (Castiglioni et al., 2021), while high-fertility bulls spermatozoa showed less susceptibility to lipid peroxidation and overexpressed transcripts associated with the reduction process compared to their low-fertile counterparts (Alvarez-Rodriguez et al., 2021; Saraf et al., 2021; Leite et al., 2022). Meanwhile, the oxidative stress index of chilled-stored boar semen showed a negative correlation with sperm motility and *in vivo* fertility (Barranco et al., 2021). The mitigation of the adverse effects of oxidative stress during preservation includes supplementation of semen extenders with various antioxidants (Amidi et al., 2016; Hosseinmardi et al., 2022; Kameni et al., 2022), providing extended protection to spermatozoa during storage. Innovative approaches for sustainable protection of spermatozoa involve utilizing nanoparticles, which show potential as antioxidants owing to their inherent redox activity. These nanoparticles aid in neutralizing and alleviating oxidative stress within extenders during (cryo)preservation (Asadi et al., 2023). Hence, the intrinsic antioxidative effects of several metal oxides (e.g., cerium, cupric, and zinc oxides), vitamin E nanoemulsions, and noble metals (e.g., gold and platinum) have been shown to offer beneficial effects, such as improved post-storage sperm motility and membrane integrity (Falchi, et al., 2018c; Valgimigli et al., 2018; Hashem and Gonzales-Bulnes, 2020; Hosseinmardi et al., 2022; Jurado-Campos et al., 2023; Khalique et al., 2024) These antioxidant nanoparticles in (cryo) preservation media improve the spermatozoa’s redox status and sperm attributes, which may positively impact pregnancy rates.

Furthermore, unilamellar liposome vesicles, prepared exosomes, and mesoporous nanoparticles can be loaded with different antioxidants for controlled release during storage. Liposomes loaded with antioxidants (e.g. quercetin, lycopene) and other lipid types (e.g., soybean lecithin) provide greater cryoprotection of spermatozoa of livestock species such as bovine (Röpke et al., 2011; Kumar et al., 2015), porcine (He et al., 2001), ovine (Luna-Orozco et al., 2019; Mafolo et al., 2020; Mortazavi et al., 2020), and equine (Pillet et al., 2012; Medina-León et al., 2019).

#### Sperm sexing

Sperm sexing is a powerful tool that selectively separates X and Y chromosome-bearing sperm cells to produce animals of predetermined sex (Yata, 2021). Flow cytometry is the modern conventional method for sperm sexing that uses lasers to differentiate between sperm genders based on their DNA content (Ugur et al., 2019). Sperm sexing offers advantages, and the commercial demand for sexed semen is increasing in cattle. However, the resulting low yield of sexed sperm numbers becomes a significant challenge, with species like pigs and horses requiring more sperm per insemination dose (Quelhas et al., 2023). A recent proteomics study has identified specific proteins of the bull sperm membrane, of which 12 and 3 were upregulated in X- and Y-bearing spermatozoa, respectively (Quelhas et al., 2021). These proteins could be potential candidates for immunoselection using nanotechnology tools like magnetic nanoparticles. A large-scale production of magnetic nanoparticles is needed, and their surface modification to target differential negative charges between X and Y sperm has successfully targeted Y sperm, leaving semen enriched with X spermatozoa (Domínguez et al., 2018).

## Conclusions and perspectives

Advanced technologies have enabled a deeper understanding of sperm biology and the processes involved in sperm phenotype and physiological status. Identifying biomarkers linked to sperm phenotype and their combination with specific nanoparticles offer new opportunities for precision breeding in livestock. This technological approach can be extended to sperm manipulation to enhance the success of various sperm biotechnologies, such as (cryo)preservation, sexing, and sperm-mediated gene transfer. Further research using nanotechnology tools is essential to improve the success rate of assisted reproduction in livestock, especially during critical seasons and instances of animal diseases.

## List of abbreviations

**ACADM**: Acyl-Coenzyme A dehydrogenase medium chain

**ADAM2**: Disintegrin and metalloproteinase domain-containing protein 2

**AI**: Artificial insemination

**AP2S1**: Adaptor related protein complex 2 subunit sigma 1

**ATP**: Adenosine triphosphate

**BAMBI**: BMP and activin membrane bound inhibitor

**BBS7**: Bardet-Biedl syndrome 7

**cAMP**: Cyclic adenosine monophosphate

**DHRS4**: Dehydrogenase/reductase 4

**DNA**: Deoxyribonucleic acid

**EAF2**: ELL associated factor 2

**ENO4**: Enolase 4

**FOS**: Fos proto-oncogene

**GLRX**: Glutaredoxin

**GPX5**: Glutathione peroxidase 5

**HSP90**: Heat shock protein 90

**HSPA8**: Heat shock protein A8

**HSPB1**: Heat shock protein family B (small) member 1

**IZUMO1**: Izumo sperm-egg fusion protein 1

**L-PDGS**: Lipocalin-type prostaglandin D synthase

**miRNA**: Micro ribonucleic acid

**mRNA**: Messenger ribonucleic acid

**MWNT**: Multi-walled carbon nanotube

**ND6**: Nicotinamide adenine dinucleotide + hydrogen dehydrogenase subunit 6

**NFATC3**: Nuclear factor of activated T cells 3

**NUDFB8**: Nicotinamide adenine dinucleotide + hydrogen: Ubiquinone Oxidoreductase Subunit B8

**OXR1**: Oxidation resistance 1

**PDIA4**: Protein disulfide isomerase family A member 4

**PE**: Polyethylene

**PEG**: Polyethylene glycol

**PTPN2**: Protein tyrosine phosphatase non-receptor type 2

**PTPRU**: Protein tyrosine phosphatase receptor type U

**QPCT**: Glutaminyl-peptide cyclotransferase

**RNA**: Ribonucleic acid

**ROS**: Reactive oxygen species

**rRNA**: Ribosomal ribonucleic acid

**SDHC**: Succinate dehydrogenase complex subunit C

**SNARE**: Soluble N-ethylmaleimide-sensitive factor attachment protein receptor

**SOF1**: Sperm-oocyte fusion required 1

**SPAC6**: Sperm acrosome associated 6

**SWCNT**: Single-walled carbon nanotube

**TMEM95**: Transmembrane protein 95

## References

[B001] Aalberts M, Stout TAE, Stoorvogel W (2014). Prostasomes: extracellular vesicles from the prostate. Reproduction.

[B002] Abedin F, Asmatulu E, Andalib MN, Kumar V, Guleria P, Ranjan  S, Dasgupta  N, Lichtfouse  E (2021). Nanotoxicology and nanoecotoxicology..

[B003] Agnihotri SK, Agrawal AK, Hakim BA, Vishwakarma AL, Narender T, Sachan R, Sachdev M (2016). Mitochondrial membrane potential (MMP) regulates sperm motility. In Vitro Cell Dev Biol Anim.

[B004] Aitken RJ, Baker MA (2008). The role of proteomics in understanding sperm cell biology. Int J Androl.

[B005] Al-Bulushi S, Manjunatha BM, Bathgate R, Rickard JP, De Graaf SP (2019). Liquid storage of dromedary camel semen in different extenders. Anim Reprod Sci.

[B006] Alpatov R, Lesch BJ, Nakamoto-Kinoshita M, Blanco A, Chen S, Stützer A, Armache KJ, Simon MD, Xu C, Ali M, Murn J, Prisic S, Kutateladze TG, Vakoc CR, Min J, Kingston RE, Fischle W, Warren ST, Page DC, Shi Y (2014). A chromatin-dependent role of the fragile X mental retardation protein FMRP in the DNA damage response. Cell.

[B007] Alvarez-Rodriguez M, Martinez CA, Roca J, Rodriguez-Martinez H (2021). mRNA expression of oxidative-reductive proteins in boars with documented different fertility can identify relevant prognostic biomarkers. Res Vet Sci.

[B008] Amann RP (2008). The cycle of the seminiferous epithelium in humans: a need to revisit?. J Androl.

[B009] Amidi F, Pazhohan A, Shabani Nashtaei M, Khodarahmian M, Nekoonam S (2016). The role of antioxidants in sperm freezing: a review. Cell Tissue Bank.

[B010] Anjum S, Ishaque S, Fatima H, Farooq W, Hano C, Abbasi BH, Anjum I (2021). Emerging applications of nanotechnology in healthcare systems: grand challenges and perspectives. Pharmaceuticals (Basel).

[B011] Ansari MS, Rakha BA, Akhter S, Ashiq M (2016). OPTIXcell improves the postthaw quality and fertility of buffalo bull sperm. Theriogenology.

[B012] Arias LS, Pessan JP, Vieira APM, De Lima TMT, Delbem ACB, Monteiro DR (2018). Iron oxide nanoparticles for biomedical applications: a perspective on synthesis, drugs, antimicrobial activity, and toxicity. Antibiotics (Basel).

[B013] Asadi Z, Safari-Faramani R, Aghaz F (2023). Effects of adding antioxidant nanoparticles on sperm parameters of non-human species after the freezing and thawing process: a systematic review and meta-analysis. Anim Reprod Sci.

[B014] Atessahin A, Numan Bucak M, Barbaros Tuncer P, Kızıl M (2008). Effects of anti-oxidant additives on microscopic and oxidative parameters of Angora goat semen following the freeze-thawing process. Small Rumin Res.

[B015] Avendaño C, Franchi A, Taylor S, Morshedi M, Bocca S, Oehninger S (2009). Fragmentation of DNA in morphologically normal human spermatozoa. Fertil Steril.

[B016] Bae JW, Yi JK, Jeong EJ, Lee WJ, Hwang JM, Kim DH, Ha JJ, Kwon WS (2022). Ras-related proteins (Rab) play significant roles in sperm motility and capacitation status. Reprod Biol.

[B017] Baker MA, Reeves G, Hetherington L, Müller J, Baur I, Aitken RJ (2007). Identification of gene products present in Triton X-100 soluble and insoluble fractions of human spermatozoa lysates using LC-MS/MS analysis. Proteomics Clin Appl.

[B018] Barranco I, Rubio CP, Tvarijonaviciute A, Rodriguez-Martinez H, Roca J (2021). Measurement of oxidative stress index in seminal plasma can predict in vivo fertility of liquid-stored porcine artificial insemination semen doses. Antioxidants.

[B019] Barwant MM, Rasool A, Mannam VK, Hussain A, Abdullah M, Memon Z, Ahamad MI, Shirani M, Maqbool KZ, Majeed M, Asif M, Khan UA, Rajput  V, Singh  A, Ghazaryan  K, Alexiou  A, Said Al-Tawaha  A (2024). Harnessing nanoomics and nanozymes for sustainable agriculture..

[B020] Bera D, Qian L, Tseng TK, Holloway PH (2010). Quantum dots and their multimodal applications: a review. Materials (Basel).

[B021] Castiglioni VC, Felipe A, Siqueira P, Bicudo LDC, De Almeida TG, Rose T, Nichi M, Diego J, Losano DA, Visitin JA (2021). Lipid peroxidation in bull semen influences sperm traits and oxidative potential of Percoll ® -selected sperm. Zygote.

[B022] Cecere JT, Dascanio JJ, McCue PM (2014). Equine reproductive procedures..

[B023] Chen Y, Cann MJ, Litvin TN, Iourgenko V, Sinclair ML, Levin LR, Buck J (2000). Soluble adenylyl cyclase as an evolutionarily conserved bicarbonate sensor. Science.

[B024] Cooper TG (2005). Cytoplasmic droplets: the good, the bad or just confusing?. Hum Reprod.

[B025] Coy P, Cánovas S, Mondéjar I, Saavedra MD, Romar R, Grullón L, Matás C, Avilés M (2008). Oviduct-specific glycoprotein and heparin modulate sperm-zona pellucida interaction during fertilization and contribute to the control of polyspermy. Proc Natl Acad Sci USA.

[B026] Dacheux JL, Dacheux F (2014). New insights into epididymal function in relation to sperm maturation. Reproduction.

[B027] DasGupta MA, Kumaresan AA, Kishor Saraf KA, Karthikkeyan GB, Keshava Prasad BTS, Kumar Modi PB, Ramesha KC, Jeyakumar SC, Manimaran AC (2021). Preliminary comparative deep metabolomic analysis of spermatozoa from zebu and crossbred cattle suggests associations between metabolites, sperm quality and fertility. Reprod Fertil Dev.

[B028] Deshpande PP, Biswas S, Torchilin VP (2013). Current trends in the use of liposomes for tumor targeting. Nanomedicine (Lond).

[B029] Díaz-Miranda EA, Maitan PP, Machado TP, Camilo BS, Lima DA, Okano DS, Penitente-Filho JM, Machado-Neves M, Oliveira LL, Guimarães SEF, Costa EP, Guimarães JD (2020). Disruption of bovine sperm functions in the presence of aplastic midpiece defect. Andrology.

[B030] Dilsiz N (2022). Hallmarks of exosomes. Future Sci OA.

[B031] Dlamini NH, Nguyen T, Gad A, Tesfaye D, Liao SF, Willard ST, Ryan PL, Feugang JM (2023). Characterization of extracellular vesicle-coupled mirna profiles in seminal plasma of boars with divergent semen quality status. Int J Mol Sci.

[B032] Dogan S, Vargovic P, Oliveira R, Belser LE, Kaya A, Moura A, Sutovsky P, Parrish J, Topper E, Memili E (2015). Sperm protamine-status correlates to the fertility of breeding bulls. Biol Reprod.

[B033] Domínguez E, Moreno-Irusta A, Castex HR, Bragulat AF, Ugaz C, Clemente H, Giojalas L, Losinno L (2018). Sperm sexing mediated by magnetic nanoparticles in donkeys, a preliminary in vitro study. J Equine Vet Sci.

[B034] Donaldson K, Aitken R, Tran L, Stone V, Duffin R, Forrest G, Alexander A (2006). Carbon nanotubes: a review of their properties in relation to pulmonary toxicology and workplace safety. Toxicol Sci.

[B035] Donaldson K, Tran CL (2002). Inflammation caused by particles and fibers. Inhal Toxicol.

[B036] Du J, Shen J, Wang Y, Pan C, Pang W, Diao H, Dong W (2016). Boar seminal plasma exosomes maintain sperm function by infiltrating into the sperm membrane. Oncotarget.

[B037] Durairajanayagam D, Rengan AK, Sharma RK, Agarwal A, Schattman  G, Esteves S,, Agarwal  A (2015). Unexplained Infertility..

[B038] Durfey CL, Swistek SE, Liao SF, Crenshaw MA, Clemente HJ, Thirumalai RVKG, Steadman CS, Ryan PL, Willard ST, Feugang JM (2019). Nanotechnology-based approach for safer enrichment of semen with best spermatozoa. J Anim Sci Biotechnol.

[B039] Elmi A, Banchelli F, Barone F, Fantinati P, Ventrella D, Forni M, Bacci ML (2018). Semen evaluation and in vivo fertility in a Northern Italian pig farm: can advanced statistical approaches compensate for low sample size? An observational study. Anim Reprod Sci.

[B040] Falchi L, Galleri G, Dore GM, Zedda MT, Pau S, Bogliolo L, Ariu F, Pinna A, Nieddu S, Innocenzi P, Ledda S (2018). Effect of exposure to CeO2 nanoparticles on ram spermatozoa during storage at 4°C for 96 hours. Reprod Biol Endocrinol.

[B041] Falchi L, Galleri G, Zedda MT, Pau S, Bogliolo L, Ariu F, Ledda S (2018). Liquid storage of ram semen for 96 hours: effects on kinematic parameters, membranes and DNA integrity, and ROS production. Livest Sci.

[B042] Falchi L, Khalil WA, Hassan M, Marei WFA (2018). Perspectives of nanotechnology in male fertility and sperm function. Int J Vet Sci Med.

[B043] Ferrigno A, Ruvolo G, Capra G, Serra N, Bosco L (2021). Correlation between the DNA fragmentation index (DFI) and sperm morphology of infertile patients. J Assist Reprod Genet.

[B044] Feugang JM, Ishak GM, Eggert MW, Arnold RD, Rivers OS, Willard ST, Ryan PL, Gastal EL (2022). Intrafollicular injection of nanomolecules for advancing knowledge on folliculogenesis in livestock. Theriogenology.

[B045] Feugang JM, Liao SF, Crenshaw MA, Clemente H, Willard ST, Ryan PL (2015). Lectin-functionalized magnetic iron oxide nanoparticles for reproductive improvement. Reprod Med Genet..

[B046] Feugang JM, Rhoads CE, Mustapha PA, Tardif S, Parrish JJ, Willard ST, Ryan PL (2019). Treatment of boar sperm with nanoparticles for improved fertility. Theriogenology.

[B047] Feugang JM, Youngblood RC, Greene JM, Fahad AS, Monroe WA, Willard ST, Ryan PL (2012). Application of quantum dot nanoparticles for potential non-invasive bio-imaging of mammalian spermatozoa. J Nanobiotechnology.

[B048] Feugang JM, Youngblood RC, Greene JM, Willard ST, Ryan PL (2015). Self-illuminating quantum dots for non-invasive bioluminescence imaging of mammalian gametes. J Nanobiotechnology.

[B049] Feugang JM (2017). Novel agents for sperm purification, sorting, and imaging. Mol Reprod Dev.

[B050] Finkelstein M, Etkovitz N, Breitbart H (2020). Ca2+ signaling in mammalian spermatozoa. Mol Cell Endocrinol.

[B051] Fitts CA, Ji N, Li Y, Tan C (2019). Exploiting exosomes in cancer liquid biopsies and drug delivery. Adv Healthc Mater.

[B052] Fraser L, Brym P, Pareek CS, Mogielnicka-brzozowska M, Paukszto Ł, Jastrze JP, Wasilewska-sakowska K, Ma A (2020). Transcriptome analysis of boar spermatozoa with different freezability using RNA-Seq. Theriogenology.

[B053] Gadella BM, Luna C (2014). Cell biology and functional dynamics of the mammalian sperm surface. Theriogenology.

[B054] Gaitskell-Phillips G, Martín-Cano FE, Ortiz-Rodríguez JM, Silva-Rodríguez A, Gil MC, Ortega-ferrusola C, Peña FJ (2021). Differences in the proteome of stallion spermatozoa explain stallion-to-stallion variability in sperm quality post-thaw†. Biol Reprod.

[B055] Gaitskell-Phillips G, Martín-Cano FE, Ortiz-Rodríguez JM, Silva-Rodríguez A, Da Silva-Álvarez E, Rojo-Domínguez P, Tapia JA, Gil MC, Ortega-Ferrusola C, Peña FJ (2021). Proteins involved in mitochondrial metabolic functions and fertilization predominate in stallions with better motility. J Proteomics.

[B056] Gallo A, Esposito MC, Tosti E, Boni R (2021). Sperm motility, oxidative status, and mitochondrial activity: exploring correlation in different species. Antioxidants.

[B057] Ganguly I, Gaur GK, Kumar S, Mandal DK, Kumar M, Singh U, Kumar S, Sharma A (2013). Differential expression of protamine 1 and 2 genes in mature spermatozoa of normal and motility impaired semen producing crossbred Frieswal (HF×Sahiwal) bulls. Res Vet Sci.

[B058] Gibbons R, Adeoya-Osiguwa SA, Fraser LR (2005). A mouse sperm decapacitation factor receptor is phosphatidylethanolamine-binding protein 1. Reproduction.

[B059] Gundogan M, Yeni D, Avdatek F, Fidan AF (2010). Influence of sperm concentration on the motility, morphology, membrane and DNA integrity along with oxidative stress parameters of ram sperm during liquid storage. Anim Reprod Sci.

[B060] Han JW, Jeong J-K, Gurunathan S, Choi Y-J, Das J, Kwon D-N, Cho S-G, Park C, Seo HG, Park J-K, Kim J-H (2016). Male- and female-derived somatic and germ cell-specific toxicity of silver nanoparticles in mouse. Nanotoxicology.

[B061] Hashem NM, Gonzales-Bulnes A (2020). State-of-the-art and prospective of nanotechnologies. Animals (Basel).

[B062] He L, Bailey JL, Buhr MM (2001). Incorporating lipids into boar sperm decreases chilling sensitivity but not capacitation potential. Biol Reprod.

[B063] Henkel RR, Schill WB (2003). Sperm preparation for ART. Reprod Biol Endocrinol.

[B064] Hill EK, Li J (2017). Current and future prospects for nanotechnology in animal production. J Anim Sci Biotechnol.

[B065] Hitit M, Memili E (2022). Sperm signatures of fertility and freezability. Anim Reprod Sci.

[B066] Hitit M, Özbek M, Ayaz-Guner S, Guner H, Oztug M, Bodu M, Kirbas M, Bulbul B, Bucak MN, Ataman MB, Memili E, Kaya A (2021). Proteomic fertility markers in ram sperm. Anim Reprod Sci.

[B067] Holstein AF, Schulze W, Davidoff M (2003). Understanding spermatogenesis is a prerequisite for treatment. Reprod Biol Endocrinol.

[B068] Holt W, Morrell J, Coward K, , Wells  D (2013). Textbook of clinical embryology..

[B069] Holt WV, Fazeli A (2015). Do sperm possess a molecular passport? Mechanistic insights into sperm selection in the female reproductive tract. Mol Hum Reprod.

[B070] Hosseinmardi M, Siadat F, Sharafi M, Roodbari NH, Hezavehei M (2022). Protective effect of cerium oxide nanoparticles on human sperm function during cryopreservation. Biopreserv Biobank.

[B071] Hungerford A, Bakos HW, Aitken RJ, Martin G (2022). Sperm cryopreservation: current status and future developments. Reprod Fertil Dev.

[B072] Inanç ME, Çi̇l B, Teki̇n K, Alemdar H, Daşkin A (2018). The combination of CASA kinetic parameters and fluorescein staining as a fertility tool in cryopreserved bull semen. Turk J Vet Anim Sci.

[B073] Jain S, Park SB, Pillai SR, Ryan PL, Willard ST, Feugang JM, Gomes AC, Sarria  MP (2018). Unraveling the safety profile of nanoscale particles and materials - from biomedical to environmental applications.

[B074] Jia B, Liang J, Lv C, Memon S, Fang Y, Wu G, Quan G (2021). The characteristics of proteome and metabolome associated with contrasting sperm motility in goat seminal plasma. Sci Rep.

[B075] Johnston DS, Wooters J, Kopf GS, Qiu Y, Roberts KP (2005). Analysis of the human sperm proteome. Ann N Y Acad Sci.

[B076] Jones RC, Robaire B., Hinton B.T. (2002). The Epididymis: From Molecules to Clinical Practice..

[B077] Jurado-Campos A, Soria-Meneses PJ, Arenas-Moreira M, Alonso-Moreno C, Rodríguez-Robledo V, Soler AJ, Garde JJ, Del Rocío Fernández-Santos M (2023). Minimizing sperm oxidative stress using nanotechnology for breeding programs in rams. J Anim Sci Biotechnol.

[B078] Kameni SL, Dongmo ABN, Tebug TT, Bomba FDT, Meutchieye F, Ngoula F (2022). Spirulina (Arthrospira platensis) extract promotes motility, microscopic, and antioxidative parameters of ram semen during refrigerated storage. BSJ Agri..

[B079] Kameni SL, Meutchieye F, Ngoula F (2021). Liquid storage of ram semen: associated damages and improvement. Open J Anim Sci.

[B080] Ka̧tska-Ksia̧zkiewicz L, Ryńska B, Gajda B, Smora̧g Z (2004). Effect of donor stimulation, frozen semen and heparin treatment on the efficiency of in vitro embryo production in goats. Theriogenology.

[B081] Khalique MA, Andrabi SMH, Majeed KA, Yousaf MS, Ahmad N, Tahir SK, Fayyaz MH, Haider MS, Naz SS, Qureshi IZ, Sulaiman S, Zaneb H, Rehman H (2024). Cerium oxide nanoparticles improve the post-thaw quality and in-vivo fertility of Beetal buck spermatozoa. Theriogenology.

[B082] Khalique MA, Rehman H, Andrabi SMH, Majeed KA, Ahmad N, Fayyaz MH, Haider MS, Naz SS, Qureshi IZ, Sulaiman S, Jammu A (2023). Antioxidant effects of zinc-oxide nanoparticles on post-thaw quality and in vivo fertility of Beetal buck spermatozoa. Small Rumin Res.

[B083] Khan GS, Tahir MZ, Zahoor MY, Rahman H, Riaz A (2024). Ameliorative effect of crocin on post-thaw quality, fertility-associated gene expression and fertilization potential of buffalo (Bubalus bubalis) bull sperm. Reprod Domest Anim.

[B084] Kherzi A, Naraud B, Stenseth EB, Johannisson A, Myromslien FD, Gaustad AH, Wilson RC, Lyle R, Morell JM, Kommisrud E, Ahmad R (2019). DNA methylation patterns vary in boar sperm cells with different levels of DNA fragmentation. BMC Genomics.

[B085] Khodaei-Motlagh M, Masoudi R, Karimi-Sabet MJ, Hatefi A (2022). Supplementation of sperm cooling medium with Zinc and Zinc oxide nanoparticles preserves rooster sperm quality and fertility potential. Theriogenology.

[B086] Kim S, Lim YT, Soltesz EG, De Grand AM, Lee J, Nakayama A, Parker JA, Mihaljevic T, Laurence RG, Dor DM, Cohn LH, Bawendi MG, Frangioni JV (2004). Near-infrared fluorescent type II quantum dots for sentinel lymph node mapping. Nat Biotechnol.

[B087] Kumar P, Saini M, Kumar D, Balhara AK, Yadav SP, Singh P, Yadav PS (2015). Liposome-based semen extender is suitable alternative to egg yolk-based extender for cryopreservation of buffalo (Bubalus bubalis) semen. Anim Reprod Sci.

[B088] Kumaresan A, Das Gupta M, Datta TK, Morrell JM (2020). Sperm DNA Integrity and male fertility in farm animals: a review. Front Vet Sci.

[B089] Kuo R, Saito E, Miller SD, Shea LD (2017). Peptide-conjugated nanoparticles reduce positive co-stimulatory expression and T cell activity to induce tolerance. Mol Ther.

[B090] Kwon W, Rahman S, Ryu D, Park Y, Pang M (2015). Increased male fertility using fertility-related biomarkers. Sci Rep.

[B091] Lacalle E, Consuegra C, Martínez CA, Hidalgo M, Dorado J, Martínez-Pastor F, Álvarez-Rodríguez M, Rodríguez-Martínez H (2022). Bicarbonate-triggered in vitro capacitation of boar spermatozoa conveys an increased relative abundance of the canonical Transient Receptor Potential Cation (TRPC) Channels 3, 4, 6 and 7 and of CatSper-γ Subunit mRNA Transcripts. Animals (Basel).

[B092] Lalancette C, Thibault C, Bachand I, Caron N, Bissonnette N (2008). Transcriptome analysis of bull semen with extreme nonreturn rate: use of suppression-subtractive hybridization to identify functional markers for fertility. Biol Reprod.

[B093] Lee B, Park Y, Pang W, Ryu D, Rahman S, Lee D, Pang M (2023). Boar fertility is controlled through systematic changes of mitochondrial protein expression during sperm capacitation. Int J Biol Macromol.

[B094] Leite RF, Losano JDA, Kawai GKV, Rui BR, Nagai KK, Castiglioni VC, Siqueira AFP, D’Avila Assumpção MEO, Baruselli PS, Nichi M (2022). Sperm function and oxidative status: effect on fertility in Bos taurus and Bos indicus bulls when semen is used for fixed-time artificial insemination. Anim Reprod Sci.

[B095] Llavanera M, Ribas-maynou J, Delgado-bermúdez A, Recuero S, Muiño R, Hidalgo CO, Tamargo C, Bonet S, Mateo-otero Y, Yeste M (2021). Sperm chromatin condensation as an in vivo fertility biomarker in bulls: a flow cytometry approach. J Anim Sci Biotechnol.

[B096] López-Úbeda R, García-Vázquez FA, Romar R, Gadea J, Muñoz M, Hunter RHF, Coy P (2015). Oviductal transcriptome is modified after insemination during spontaneous ovulation in the sow. PLoS One.

[B097] Lu CH, Lee RKK, Hwu YM, Chu SL, Chen YJ, Chang WC, Lin SP, Li SH (2011). SERPINE2, a serine protease inhibitor extensively expressed in adult male mouse reproductive tissues, may serve as a murine sperm decapacitation factor. Biol Reprod.

[B098] Lucca MS, Dal R, Gianluppi F, Paula A, Mellagi G, Pandolfo Bortolozzo F, Wentz I, Da R, Ulguim R (2021). Effects of the classification of boars according to progressive sperm motility and the extender type on the reproductive performance of a single fixed-time insemination. Theriogenology.

[B099] Luna-Orozco JR, González-Ramos MA, Calderón-Leyva G, Gaytán-Alemán LR, Arellano-Rodríguez MA, Ángel-García O, Véliz-Deras FG (2019). Comparison of different diluents based on liposomes and egg yolk for ram semen cooling and cryopreservation. IJVR.

[B100] Mafolo KS, Pilane CM, Chitura T, Nedambale TL (2020). Use of phosphatidylcholine in Tris-based extender with or without egg yolk to freeze Bapedi ram semen. S Afr J Anim Sci.

[B101] Mateo-Otero Y, Madrid-gambin F, Llavanera M, Gomez-gomez A, Haro N, Pozo OJ, Yeste M (2023). Sperm physiology and in vitro fertilising ability rely on basal metabolic activity: insights from the pig model. Commun Biol.

[B102] Medina-León AZ, Domínguez-mancera B, Cazalez-penino N, Cervantes-Acosta P, Jácome-Sosa E, Romero-Salas D, Barrientos-Morales M (2019). Cryopreservation of horse semen with a liposome and trehalose added extender. Austral J Vet Sci.

[B103] Meles DK, Mustofa I, Hariadi M, Wurlina W, Susilowati S, Amaliya A, Suparto S, Rimayanti R (2022). The enriched Y-bearing sperm combined with delayed fixed-time artificial insemination for obtaining male Simmental crossbred offspring. Vet World.

[B104] Menezes EB, Velho ALC, Santos F, Dinh T, Kaya A, Topper E, Moura AA, Memili E (2019). Uncovering sperm metabolome to discover biomarkers for bull fertility. BMC Genomics.

[B105] Mo R, Jiang T, Gu Z (2014). Recent progress in multidrug delivery to cancer cells by liposomes. Nanomedicine (Lond).

[B106] Mokarizadeh A, Rezvanfar M, Dorostkar K, Abdollahi M (2013). Mesenchymal stem cell derived microvesicles: trophic shuttles for enhancement of sperm quality parameters. Reprod Toxicol.

[B107] Montecalvo A, Larregina AT, Shufesky WJ, Stolz DB, Sullivan MLG, Karlsson JM, Baty CJ, Gibson GA, Erdos G, Wang Z, Milosevic J, Tkacheva OA, Divito SJ, Jordan R, Lyons-Weiler J, Watkins SC, Morelli AE (2012). Mechanism of transfer of functional microRNAs between mouse dendritic cells via exosomes. Blood.

[B108] Mortazavi S, Eslami M, Farrokhi-ardabili F (2020). Comparison of different carrier-compounds and varying concentrations of oleic acid on freezing tolerance of ram spermatozoa in tris-citric acid-egg yolk plasma semen diluent. Anim Reprod Sci.

[B109] NNI (2024). NNI.

[B110] Neto FTL, Bach PV, Najari BB, Li PS, Goldstein M (2016). Spermatogenesis in humans and its affecting factors. Semin Cell Dev Biol.

[B111] Odhiambo JF, Dejarnette JM, Geary TW, Kennedy CE, Suarez SS, Sutovsky P (2014). Increased conception rates in beef cattle inseminated with nanopurified bull downloaded from downloaded from. Biol Reprod.

[B112] Paciotti GF, Kingston DGI, Tamarkin L (2006). Colloidal gold nanoparticles: a novel nanoparticle platform for developing multifunctional tumor-targeted drug delivery vectors. Drug Dev Res.

[B113] Peddinti D, Nanduri B, Kaya A, Feugang JM, Burgess SC, Memili E (2008). Comprehensive proteomic analysis of bovine spermatozoa of varying fertility rates and identification of biomarkers associated with fertility. BMC Syst Biol.

[B114] Pessoa ER, Roger Vasconcelos F, de Oliveira Paula‐Marinho S, de Menezes Daloso D, Damasceno Guerreiro D, Matias Martins JA, Gomes-Filho E, Alencar Moura A (2023). Metabolomic profile of seminal plasma from Guzerá bulls (Bos indicus) with contrasting sperm freezability phenotypes. Reprod Domest Anim.

[B115] Piehl LL, Fischman ML, Hellman U, Cisale H, Miranda PV (2013). Boar seminal plasma exosomes: effect on sperm function and protein identification by sequencing. Theriogenology.

[B116] Pillet E, Labbe C, Batellier F, Duchamp G, Beaumal V, Anton M, Desherces S, Schmitt E, Magistrini M (2012). Liposomes as an alternative to egg yolk in stallion freezing extender. Theriogenology.

[B117] Purdy PH, Graham JK, Wolkers W, , Oldenhof  H (2015). Cryopreservation and freeze-drying protocols..

[B118] Qamar AY, Fang X, Kim MJ, Cho J (2019). Improved post-thaw quality of canine semen after treatment with exosomes from conditioned medium of adipose-derived mesenchymal stem cells. Animals (Basel).

[B119] Quelhas J, Pinto-Pinho P, Lopes G, Rocha A, Pinto-Leite R, Fardilha M, Colaço B, Feugang J, Chapwanya A, Bianchi M, Alves R (2023). Sustainable animal production: exploring the benefits of sperm sexing technologies in addressing critical industry challenges. Front Vet Sci.

[B120] Quelhas J, Santiago J, Matos B, Rocha A, Lopes G, Fardilha M (2021). Bovine semen sexing: sperm membrane proteomics as candidates for immunological selection of X‐ and Y‐chromosome‐bearing sperm. Vet Med Sci.

[B121] Rodríguez-Martínez H, Kvist U, Ernerudh J, Sanz L, Calvete JJ (2011). Seminal plasma proteins: what role do they play?. Am J Reprod Immunol.

[B122] Röpke T, Oldenhof H, Leiding C, Sieme H, Bollwein H, Wolkers WF (2011). Liposomes for cryopreservation of bovine sperm. Theriogenology.

[B123] Saadeldin IM, Khalil WA, Alharbi MG, Lee SH (2020). The Current Trends in Using Nanoparticles, Liposomes, and Exosomes for Semen Cryopreservation. Animals (Basel).

[B124] Sapanidou V, Tsantarliotou MP, Lavrentiadou SN (2023). A review of the use of antioxidants in bovine sperm preparation protocols. Anim Reprod Sci.

[B125] Saraf KK, Kumaresan A, Sinha MK, Datta TK (2021). Spermatozoal transcripts associated with oxidative stress and mitochondrial membrane potential differ between high‐ and low‐fertile crossbred bulls. Andrologia.

[B126] Saravia F, Wallgren M, Johannisson A, Calvete JJ, Sanz L, Peña FJ, Roca J, Rodríguez-Martínez H (2009). Exposure to the seminal plasma of different portions of the boar ejaculate modulates the survival of spermatozoa cryopreserved in MiniFlatPacks. Theriogenology.

[B127] Sellem E, Broekhuijse MLWJ, Chevrier L, Camugli S, Schmitt E, Schibler L, Koenen EPC (2015). Use of combinations of in vitro quality assessments to predict fertility of bovine semen. Theriogenolog..

[B128] Selokar NL, Dua S, Kumar D, Sharma B, Saini M, Ghorbanpour  M, Bhargava  P, Varma  A, Choudhary  D (2020). Biogenic nano-particles and their use in agro-ecosystem..

[B129] Sharma R, Agarwal A, Zini A, Agarwal A (2011). Sperm chromatin..

[B130] Sharma U, Conine CC, Shea JM, Boskovic A, Derr AG, Bing XY, Belleannee C, Kucukural A, Serra RW, Sun F, Song L, Carone BR, Ricci EP, Li XZ, Fauquier L, Moore MJ, Sullivan R, Mello CC, Garber M, Rando OJ (2016). Biogenesis and function of tRNA fragments during sperm maturation and fertilization in mammals. Science.

[B131] Siu KK, Serrão VHB, Ziyyat A, Lee JE (2021). The cell biology of fertilization: gamete attachment and fusion. J Cell Biol.

[B132] Soggiu A, Piras C, Hussein HA, De Canio M, Gaviraghi A, Galli A, Urbani A, Bonizzi L, Roncada P (2013). Unravelling the bull fertility proteome. Mol Biosyst.

[B133] Song C, Chang L, Wang B, Zhang Z, Wei Y, Dou Y, Qi K, Yang F, Li X, Li X, Wang K, Qiao R, Han X (2023). Seminal plasma metabolomics analysis of differences in liquid preservation ability of boar sperm. J Anim Sci.

[B134] Song C, Zhang Z, Wei Y, Dou Y, Qi K, Li X, Yang F, Li X, Wang K, Qiao R, Han X (2024). Proteomic analysis of boar sperm with differential ability of liquid preservation at 17°C. Theriogenology.

[B135] Sostaric E, Dieleman SJ, Van De Lest CHA, Colenbrander B, Vos PLAM, Garcia-Gil N, Gadella BM (2008). Sperm binding properties and secretory activity of the bovine oviduct immediately before and after ovulation. Mol Reprod Dev.

[B136] Souza ET, Silva CV, Augusto B, Travençolo N, Geraldo B, Emílio M (2018). Sperm chromatin alterations in fertile and subfertile bulls. Reprod Biol.

[B137] Staub C, Johnson L (2018). Review: spermatogenesis in the bull. Animal.

[B138] Suchocki T, Szyda J (2015). Genome-wide association study for semen production traits in Holstein-Friesian bulls. J Dairy Sci.

[B139] Sui H, Sheng M, Luo H, Liu G, Meng F, Cao Z, Zhang Y (2023). Characterization of freezability-associated metabolites in boar semen. Theriogenology.

[B140] Suk JS, Xu Q, Kim N, Hanes J, Ensign LM (2016). PEGylation as a strategy for improving nanoparticle-based drug and gene delivery HHS Public Access Graphical abstract. Adv Drug Deliv Rev.

[B141] Sullivan R, Saez F, Girouard J, Frenette G (2005). Role of exosomes in sperm maturation during the transit along the male reproductive tract. Blood Cells Mol Dis.

[B142] Sun P, Zhang G, Xian M, Zhang G, Wen F, Hu Z, Hu J (2023). Proteomic analysis of frozen–thawed spermatozoa with different levels of freezability in dairy goats. Int J Mol Sci.

[B143] Sun T, Zhang YS, Pang B, Hyun DC, Yang M, Xia Y (2014). Engineered nanoparticles for drug delivery in cancer therapy. Angew Chem Int Ed Engl.

[B144] Sutovsky P, Hamilton LE, Zigo M, Ortiz DME, Assumpção A, Jones A, Tirpak F, Agca Y, Kerns K, Sutovsky M (2024). Biomarker-based human and animal sperm phenotyping. Biol Reprod.

[B145] Sutovsky P, Lovercamp K (2010). Molecular markers of sperm quality. Soc Reprod Fertil Suppl.

[B146] Swelum AA-A, Saadeldin IM, Ba-Awadh H, Al-Mutary MG, Moumen AF, Alowaimer AN, Abdalla H (2019). Efficiency of Commercial Egg Yolk-Free and Egg Yolk-Supplemented Tris-Based Extenders for Dromedary Camel Semen Cryopreservation. Animals (Basel).

[B147] Szczęch M, Szczepanowicz K (2020). Polymeric core-shell nanoparticles prepared by spontaneous emulsification solvent evaporation and functionalized by the layer-by-layer method. Nanomaterials (Basel).

[B148] Talevi R, Gualtieri R (2010). Molecules involved in sperm-oviduct adhesion and release. Theriogenology.

[B149] Tardif S, Dubé C, Chevalier S, Bailey JL (2001). Capacitation is associated with tyrosine phosphorylation and tyrosine kinase-like activity of pig sperm proteins. Biol Reprod.

[B150] Torres MA, Pedrosa AC, Novais FJ, Alkmin DV, Cooper BR, Yasui GS, Fukumasu H, Machaty Z, Andrade AFCD (2022). Metabolomic signature of spermatozoa established during holding time is responsible for differences in boar sperm†. Biol Reprod.

[B151] Touré A (2019). Importance of slc26 transmembrane anion exchangers in sperm post-testicular maturation and fertilization potential. Front Cell Dev Biol.

[B152] Travis AJ, Kopf GS (2002). The role of cholesterol efflux in regulating the fertilization potential of mammalian spermatozoa. J Clin Invest.

[B153] Trimeche A, Yvon JM, Vidament M, Palmer E, Magistrini M (1999). Effects of glutamine, proline, histidine and betaine on post-thaw motility of stallion spermatozoa. Theriogenology.

[B154] Troedsson MHT, Desvousges A, Alghamdi AS, Dahms B, Dow CA, Hayna J, Valesco R, Collahan PT, Macpherson ML, Pozor M, Buhi WC (2005). Components in seminal plasma regulating sperm transport and elimination. Anim Reprod Sci.

[B155] Ugur MR, Dinh T, Hitit M, Kaya A, Topper E, Didion B, Memili E (2020). Amino acids of seminal plasma associated with freezability of bull sperm. Front Cell Dev Biol.

[B156] Ugur MR, Saber Abdelrahman A, Evans HC, Gilmore AA, Hitit M, Arifiantini RI, Purwantara B, Kaya A, Memili E (2019). Advances in Cryopreservation of Bull Sperm. Front Vet Sci.

[B157] Ul Haq Z, Hamadani H, Khan AA, Ganai AM, Beigh YA, Gull Sheikh G, Farooq J, Ahmad Ganai I, Ahmad SM, Sheikh FA, , Majeed S, , Beigh MA (2023). Interaction of nanomaterials with living cells..

[B158] Valgimigli L, Baschieri A, Amorati R (2018). Antioxidant activity of nanomaterials. J Mater Chem B Mater Biol Med.

[B159] Vicente-Fiel S, Palacín I, Santolaria P, Fantova E, Quintín-casorrán FJ, Sevilla-Mur E, Yániz JL (2014). In vitro assessment of sperm quality from rams of high and low field fertility. Anim Reprod Sci.

[B160] Virlan MJR, Miricescu D, Radulescu R, Sabliov CM, Totan A, Calenic B, Greabu M (2016). Organic nanomaterials and their applications in the treatment of oral diseases. Molecules.

[B161] Wang L, Zhao W, Tan W (2008). Bioconjugated silica nanoparticles: development and applications. Nano Res.

[B162] Wang Y, Quinsaat JEQ, Ono T, Maeki M, Tokeshi M, Isono T, Tajima K, Satoh T, Sato S, Miura Y, Yamamoto T (2020). Enhanced dispersion stability of gold nanoparticles by the physisorption of cyclic poly(ethylene glycol). Nat Commun.

[B163] Weng J, Ren J (2006). Luminescent Quantum Dots: A Very Attractive and Promising Tool in Biomedicine. Curr Med Chem.

[B164] Wiebke M, Hensel B, Nitsche-Melkus E, Jung M, Schulze M (2022). Cooled storage of semen from livestock animals (part I): boar, bull, and stallion. Anim Reprod Sci.

[B165] Xu C, Nam J, Hong H, Xu Y, Moon JJ (2019). Positron emission tomography-guided photodynamic therapy with biodegradable mesoporous silica nanoparticles for personalized cancer immunotherapy. ACS Nano.

[B166] Yang C, Guo W-B, Zhang W-S, Bian J, Yang J-K, Zhou Q-Z, Chen M-K, Peng W, Qi T, Wang C-Y, Liu C-D (2017). Comprehensive proteomics analysis of exosomes derived from human seminal plasma. Andrology.

[B167] Yang W, Liang H, Ma S, Wang D, Huang J (2019). Gold nanoparticle based photothermal therapy: development and application for effective cancer treatment. Sust Mat Tech..

[B168] Yata VK, Yata VK (2021). Microfluidics for assisted reproduction in animals..

[B169] Yeste M (2016). Sperm cryopreservation update: Cryodamage, markers, and factors affecting the sperm freezability in pigs. Theriogenology.

[B170] Yoon S-J, Kwon W-S, Rahman MS, Lee J-S, Pang M-G (2015). A novel approach to identifying physical markers of cryo-damage in bull spermatozoa. PLoS One.

[B171] Yuan Y, Yu M, Chen L, Ren X, Qu Y, Shari A, Li G (2023). Comparative analysis of different metabolites in semen of Guanzhong dairy goats with different motility rates. Theriogenology.

[B172] Yuan Y, Wang G, Zou J, Zhang Y, Li D, Yu M, Chen L, Li G (2023). Study on comparative analysis of differential metabolites in Guanzhong dairy goat semen before and after freezing. Theriogenology.

[B173] Zeng F, Chen Y, Guo C, Li C, Wei H, Li L, Meng L, Zhang S (2021). Analysis of differentially abundant proteins related to boar fertility in seminal plasma using iTRAQ-based quantitative proteomics. J Proteomics.

[B174] Zhang H, Liu H, Kataoka S, Kinukawa M, Uchiyama K, Kambe J, Watanabe G, Jin W, Nagaoka K (2021). L-amino acid oxidase 1 in sperm is associated with reproductive performance in male mice and bulls. Biol Reprod.

[B175] Zhang Y, Yuan W, Liu Y, Liu Y, Liang H, Xu Q, Liu Z, Weng X (2023). Plasma membrane lipid composition and metabolomics analysis of Yorkshire boar sperms with high and low resistance to cryopreservation. Theriogenology.

[B176] Zhao Y, Qin J, Sun J, He J, Sun Y, Yuan R, Li Z (2024). Motility-related microRNAs identified in pig seminal plasma exosomes by high-throughput small RNA sequencing. Theriogenology.

[B177] Zhu W, Zhang Y, Ren C, Cheng X, Chen J, Ge Z, Sun ZP, Zhuo X, Sun FF, Chen YL, Jia XJ, Zhang Z (2020). Identification of proteomic markers for ram spermatozoa motility using a tandem mass tag (TMT) approach. J Proteomics.

[B178] Zielińska A, Carreiró F, Oliveira AM, Neves A, Pires B, Nagasamy Venkatesh D, Durazzo A, Lucarini M, Eder P, Silva AM, Santini A, Souto EB (2020). Polymeric nanoparticles: production, characterization, toxicology and ecotoxicology. Molecules.

